# Association between ankle-brachial blood pressure index with all-cause and cardiovascular mortality in adults without arterial stiffness

**DOI:** 10.1186/s12877-023-04332-z

**Published:** 2023-10-09

**Authors:** Zhe Meng, Yaohui Jiang, Chang Xu, Huifen Zheng, Haiyu Li

**Affiliations:** Department of Cardiology, The First Affiliated Hospital of Zhengzhou University, Zhengzhou, China

**Keywords:** Ankle-brachial blood pressure index, All-cause mortality, Cardio-cerebrovascular mortality, Peripheral arterial disease, Arterial calcification

## Abstract

**Purpose:**

To explore the relationship between ankle-brachial blood pressure index (ABPI) and all-cause or cardiovascular mortality in adults without arterial stiffness.

**Methods:**

A total of 6784 participants without arterial stiffness were enrolled from National Health and Nutrition Examination Survey 1999–2004. The hazard ratio (HR) and 95% confidence interval (CI) of ABPI associating with the risk of all-cause and cardiovascular mortality was calculated by Cox proportional regression models adjusted for demographic and traditional risk factors. Dose-response relationship was explored with restricted cubic spines.

**Results:**

After an average follow-up of 12.1 years, 1844 all-cause deaths and 299 cardiovascular deaths occurred. Compared with the lowest ABPI quartile, the second quartile was associated with the lowest risk of all-cause mortality (HR 0.89, 95%CI 0.79–0.98; p = 0.036) and cardiovascular mortality (HR 0.75, 95%CI 0.56–0.98; p = 0.048). Besides, dose-response analysis revealed that ABPI was nonlinearly correlated to all-cause mortality (p for nonlinearity < 0.001) and linearly correlated to cardiovascular mortality (p for nonlinearity = 0.459).

**Conclusions:**

The relationship between ABPI and all-cause and cardiovascular mortality followed a L-shape curve. A lower ABPI was independently associated with an increased risk of all-cause and cardiovascular mortality in adults without arterial stiffness.

## Introduction

In the elderly, the spectrum of cause of death is diverse, which involves cardiovascular diseases, diabetes, and cerebrovascular diseases. Arterial stiffness refers to the buildup of plaque within the arteries, causing them to narrow and harden [1]. It affects multiple organs, including the heart, brain, kidneys, and lower extremities [2]. Therefore, it is a common underlying condition that contributes to various causes of death in this population [3]. Various indexes and assessment tools can be utilized for prediction of arterial stiffness, including ankle-brachial blood pressure index (ABPI) [4], cardio-ankle vascular index (CAVI) [5] and brachial-ankle pulse wave velocity (BaPWV) [6].

ABPI is a non-invasive tool used to evaluate vascular stiffness. It was calculated as the ratio of the highest systolic pressure in tibial artery to the highest systolic pressure in brachial artery. Previous studies showed that an ABPI > 1.3 is a reliable marker of arterial stiffness [7] and an ABPI < 0.9 was diagnosed as peripheral arterial disease [8]. Patients with peripheral arterial disease have higher risk of coronary heart diseases and stoke [9], and is a predictor of future cardiovascular events and mortality [10]. Therefore, ABPI is a strong predictor of cardiovascular disease morbidity and mortality [11].

Among generally healthy, community-dwelling adults, arterial stiffness is associated with higher cardiovascular mortality [12]. However, the association between ABPI and all-cause and cardiovascular mortality in adults without vascular stiffness (as estimated by ABPI > 1.3) has not been examined. So, the purpose of this study is to investigate the predictive value of ABPI in adults without arterial stiffness.

## Methods

### Study population

The study used data from National Health and Nutrition Examination Survey (NHANES) between the periods of 1999 to 2004, a nationwide survey conducted by the National Center of Health Statistics (NCHS). The survey was designed to assess the health and nutritional status of the non-institutionalized US population by a stratified and multistage sampling design. Standardized questionnaires were administered in the home, followed by a detailed physical examination and blood specimens at a mobile examination center.

After excluding participants with missing data on ABPI (n = 1574), there were 7571 participants with completed records of ABPI. Prior studies showed that an ABPI > 1.3 is a reliable marker of arterial stiffness [13] and an ABPI < 0.9 was diagnosed as peripheral arterial disease [14]. Therefore, we excluded participants with ABPI > 1.3 (n = 331) and ABPI < 0.9 (n = 447), as well as unavailable data on mortality (n = 9). Finally, a total of 6784 participants were enrolled in the study. The study was approved by the institutional review board of NCHS and all participants provided written informed consent.

### Exposure variable and outcomes

All blood pressure measurements were performed in a standardized fashion at the mobile examination centers. Systolic pressure was measured on the both arms (brachial artery) and both ankles (posterior tibial arteries) using an 8-MHz Doppler probe. Each pressure was measured twice for participants aged 40–59 years and once for participants aged > 60 yrs to reduce the time for this component in that age group. The ABPI was automatically calculated by dividing the mean systolic blood pressure in the ankle by in the arm (Parks Mini-Laboratory IV, Model 3100).

The primary outcome was all-cause mortality while the secondary outcomes included death from cardiovascular mortality, which was obtained by linkage to the National Death Index by 31 December 2015.

### Covariates collection

Questionnaires data included age, gender and ethnicity. A diagnosis of hypertension was assigned if the subject had a history of hypertension or blood pressure ≥ 140/90 mmHg or taking anti-hypertensive medications. A diagnosis of diabetes was assigned if the subject reported a physician.

diagnosis of diabetes or taking hypoglycemic medications or if fasting plasma glucose was ≥ 7.0 mmol/L. The family poverty income ratio (PIR) is a ratio of family income to poverty threshold. PIR was used to create three categories of income status, low (PIR < 1), mid (1 ~ 3) and high (> 3), as an indication of socioeconomic status based on eligibility for receiving benefits via the Supplemental Nutrition Assistance Program. Smoker was defined as the individual who reported to have smoked more than 100 cigarettes in his live. The physical activity was categorized into three degrees over past 30 days. Vigorous activities were defined as that cause heavy sweating or large increases in breathing or heart rate. Moderate activities were defined as that cause only light sweating or a slight to moderate increase in breathing or heart rate. Height and weight were measured by physical examination by trained personnel and body mass index (BMI) is calculated as weight (kg)/(height (m))^2. Multiple imputation using predictive mean matching (PMM) was performed for covariates with missing values.

### Statistical analysis

Continuous variables were presented as the mean (standard deviation), and categorical variables were presented as numbers (percentage). Differences between groups were explored by one-way analysis of variance, or chi-square tests. Multivariate Cox proportional hazards regression models were used to estimate hazard ratios (HRs) and 95% confidence interval (CI) for all-cause, and cardiovascular mortality. Model 1 was not adjusted. Model 2 was adjusted for age, gender and ethnicity. Model 3 was adjusted for age, gender, ethnicity, education level, PIR, body mass index (BMI), smoker, physical activity, hypertension and diabetes. Sensitive analysis was performed to explore the interactions (hypertension and diabetes) modifying the relationship. Dose-response analysis was performed by restricted cubic splines. All statistical analyses were performed using R version 3.6, and P < 0.05 was considered as statistically significant.

## Results

6784 participants were included in the present study, of which there were 3390 (51%) male, and mean age was 59.5 years old. During the average 12.1 years of follow-up, 1844 all-cause deaths and 299 cardiovascular deaths occurred. The baseline characteristics of population were presentenced according to ABPI quartiles in Table 1. Individuals with lower ABPI levels were older and more female, as well as had more percentage of hypertension and diabetes.

**Table 1:** Characteristics of the study population according to the ABPI quartiles

Variables	Overall (n = 6784)	Q1 (n = 1705)	Q2 (n = 1737)	Q3 (n = 1711)	Q4 (n = 1631)	*P* value
ABPI	1.12 (0.09)	1.00 (0.04)	1.09 (0.02)	1.15 (0.02)	1.23 (0.03)	< 0.001
Male (%)	3390 (51.0)	610 (35.8)	805 (46.3)	962 (56.2)	1013 (62.1)	< 0.001
Age, years	59.5 (12.9)	63.4 (12.9)	59.4 (12.6)	58.7 (12.5)	56.9 (12.3)	< 0.001
Ethnicity (%)						< 0.001
Non-Hispanic white	3625 (53.9)	807 (47.3)	884 (50.9)	970 (56.7)	964 (59.1)	
Non-Hispanic black	1232 (18.2)	440 (25.8)	352 (20.3)	256 (15.0)	184 (11.3)	
Mexican American	1441 (21.2)	335 (19.6)	370 (21.3)	363 (21.2)	373 (22.9)	
Others	486 (7.2)	123 (7.2)	131 (7.5)	122 (7.1)	110 (6.7)	
Education (%)						< 0.001
Less than high school	2261 (33.3)	660 (38.7)	578 (33.3)	535 (31.3)	488 (29.9)	
High school	1592 (23.5)	397 (23.3)	457 (26.3)	387 (22.6)	351 (21.5)	
More than high school	2931 (43.2)	648 (38.0)	702 (40.4)	789 (46.1)	792 (48.6)	
PIR (%)						< 0.001
< 1	1052 (15.5)	322 (18.9)	286 (16.5)	222 (13.0)	222 (13.6)	
1 ~ 3	2832 (41.7)	808 (47.4)	718 (41.3)	679 (39.7)	627 (38.4)	
> 3	2900 (42.7)	575 (33.7)	733 (42.2)	810 (47.3)	782 (47.9)	
BMI, kg/m2	28.4 (5.6)	28.4 (5.9)	28.5 (5.6)	28.4 (5.4)	28.4 (5.5)	0.99
Smoker (%)	1937 (28.6)	557 (32.7)	544 (31.3)	465 (27.2)	371(22.7)	< 0.001
Activity (%)						< 0.001
Vigorous	3801 (56.0)	1090 (63.9)	984 (56.6)	906 (53.0)	821 (50.3)	
Moderate	2459 (36.2)	470 (27.6)	608 (35.0)	676 (39.5)	705 (43.2)	
Inactive	524 (7.7)	145 (8.5)	145 (8.3)	129 (7.5)	105 (6.4)	
Hypertension	2216 (32.7)	777 (45.6)	602 (34.7)	460 (26.9)	377 (23.1)	< 0.001
Diabetes	1141 (16.8)	343 (20.1)	286 (16.5)	276 (16.1)	236 (14.5)	< 0.001
Triglycerides, mg/dL						
Cholesterol, mg/dL	208 (40.8)	211 (41.7)	210 (40.1)	207(40.4)	203 (40.6)	< 0.001
HbA1c, %	7.36 (1.84)	7.35 (1.73)	7.40 (1.83)	7.34 (1.83)	7.31 (1.73)	0.821
SBP, mmHg	133 (21)	140 (24)	133 (21)	129 (19)	126 (17)	< 0.001
DBP, mmHg	72 (14)	71 (16)	73 (14)	73 (13)	72 (13)	< 0.001
CAD, %	207 (3.1)	42 (2.5)	21 (1.2)	60 (3.5)	84 (5.2)	0.015
Stroke, %	174 (2.6)	48 (2.8)	9 (0.5)	33 (1.9)	84 (5.2)	0.025
CKD, %	174 (2.6)	15 (0.9)	27 (1.5)	87 (5.0)	45 (2.8)	0.017
Antihypertensive drugs	1284 (18.6)	456 (26.7)	336 (19.3)	243 (14.2)	229(14.0)	0.002
Lipid-lowering drugs	1158 (17.1)	318 (18.6)	289 (16.6)	289 (16.6)	262 (16.1)	0.112

ABPI, ankle-brachial pressure index; PIR, poverty income ratio; BMI, body mass index; SBP, systolic blood pressure; DBP, diastolic blood pressure; CAD, coronary artery disease; CKD, chronic kidney disease

As for all-cause mortality shown in Table 2, when compared with the lowest quartile, the HR and 95% CI of the second quartile was 0.66 (0.59–0.75; p < 0.001) in unadjusted Model 1, 0.80 (0.71–0.91; p < 0.001) in partly-adjusted Model 2, and 0,89 (0.79–0.98; p = 0.036) in fully-adjusted model 3.

**Table 2:** Association of ABPI quartile with all-cause mortality

	Q1		Q2		Q3		Q4	
	HR	P	HR (95% CI)	P	HR (95% CI)	P	HR (95% CI)	P
Model 1	ref		0.66 (0.59, 0.75)	< 0.001	0.61 (0.53, 0.69)	< 0.001	0.53 (0.47, 0.61)	< 0.001
Model 2	ref		0.80 (0.71, 0.91)	< 0.001	0.78 (0.69, 0.89)	< 0.001	0.76 (0.66, 0.87)	< 0.001
Model 3	ref		0.89 [0.79, 0.98]	0.036	0.93 [0.82, 1.05]	0.276	0.95 [0.83, 1.08]	0.705

Model 1 was unadjusted

Model 2 was adjusted for age, gender and ethnicity

Model 3 was adjusted for gender, age, ethnicity, education, PIR, BMI, smoker, activity, hypertension, diabetes, lipid profile, HbA1c, SBP, DBP, CKD, CAD, stroke, lipid-lowering drugs and antihypertensive drugs

HR, hazard ratio; CI, confidence interval

Table 3 showed the association of ABPI quartile with cardiovascular mortality. Only the second quartile was significantly associated with mortality across three models. After adjusted for demographics, lifestyles and comorbidities, the second quartile was independently associated with 0.75-fold (0.56–0.98; p = 0.048) risk of cardiovascular mortality.

**Table 3:** Association of ABPI quartile with cardiovascular mortality

	Q1		Q2		Q3		Q4	
	HR	P	HR (95% CI)	P	HR (95% CI)	P	HR (95% CI)	P
Model 1	ref		0.67 [0.49, 0.91]	0.010	0.74 [0.54, 1.00]	0.050	0.70 [0.50, 0.96]	0.029
Model 2	ref		0.69 [0.51, 0.94]	0.020	0.71 [0.52, 0.97]	0.033	0.67 [0.48, 0.94]	0.021
Model 3	ref		0.75 [0.56, 0.98]	0.048	0.79 [0.58, 1.06]	0.098	0.75 [0.55, 1.05]	0.095

Model 1 was unadjusted

Model 2 was adjusted for age, gender and ethnicity

Model 3 was adjusted for gender, age, ethnicity, education, PIR, BMI, smoker, activity, hypertension, diabetes, lipid profile, HbA1c, SBP, DBP, CKD, CAD, stroke, lipid-lowering drugs and antihypertensive drugs

HR, hazard ratio; CI, confidence interval

What’s more, sensitive analysis was used to explore the interactors (diabetes and hypertension) between ABPI and all-cause mortality. As indicated in Table 4, the association was consistent across the presence/absence of diabetes or hypertension.

**Table 4:** Sensitive analysis of association of ABPI with all-cause mortality

	Q1	Q2	Q3	Q4	P for interaction
**Diabetes**					0.846
No	Ref	0.89 [0.77, 1.03]	0.94 [0.79, 1.07]	0.93 [0.82, 1.12]	
Yes	Ref	0.87 [0.68, 1.13]	0.96 [0.74, 1.23]	0.87 [0.62, 1.09]	
**Hypertension**					0.185
No	Ref	0.83 [0.78, 1.04]	0.98 [0.76, 1.02]	0.85 [0.76, 1.04]	
Yes	Ref	0.92 [0.63, 1.07]	1.05 [0.84, 1.42]	1.09 [0.78, 1.39]	

**Fig. 1:**
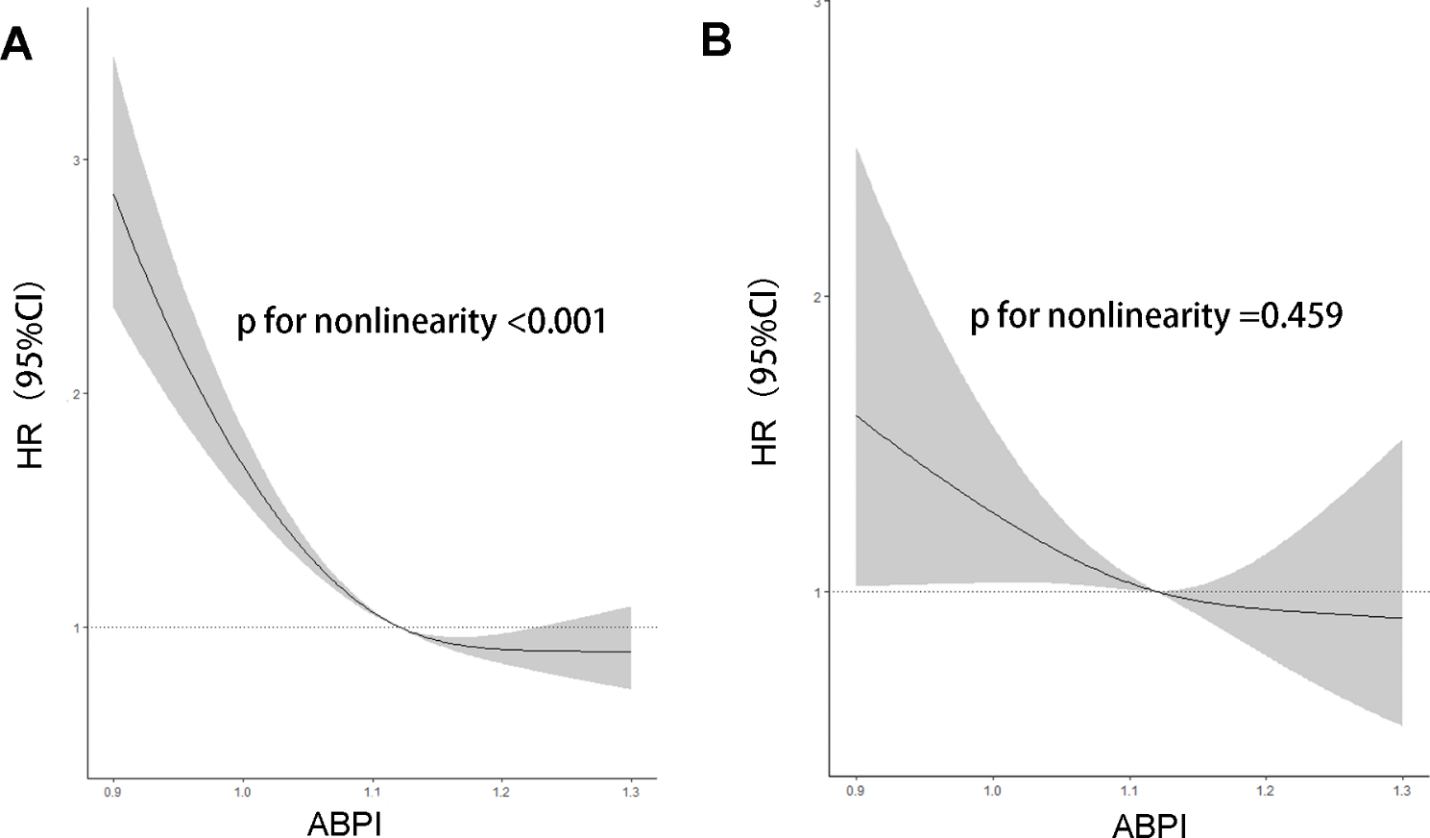
The dose-response relationship between the ABPI with all-cause mortality (**A**) and cardiovascular mortality (**B**)

Restricted cubic spline regressions were used to explore the dose-response relationship between ABPI and mortality. The results suggested that ABPI was nonlinearly correlated to all-cause mortality (p for nonlinearity < 0.001) and linearly correlated to cardiovascular mortality (p for nonlinearity = 0.459). Both the relationships were L shaped, namely lower levels of ABPI were associated with an increased risk of all-cause and cardiovascular mortality (Fig. 1).

## Discussion

In our study, we found that the second quartile was associated with the lowest risk of all-cause mortality (HR 0.89, 95%CI 0.79–0.98; p = 0.036) and cardiovascular mortality (HR 0.75, 95%CI 0.56–0.98; p = 0.048) in adults without arterial stiffness. The relationship between ABPI and all-cause and cardiovascular mortality followed a L-shape curve.

A magnitude of studies have reported that a lower ABPI was associated with an increased risk of diabetes [15], cardiovascular diseases [16], and poor nutritional status [17]. Besides, a high ABPI > 1.3 indicates the calcification of the incompressible vessels reflecting arterial stiffness, which has been associated with increased risk of cardiovascular morbidity and mortality [18]. In consistent with previous studies, our results also demonstrated that the lower ABPI quartile was associated with the highest risk of all-cause mortality and cardiovascular mortality in adults without arterial stiffness. ABPI was nonlinearly related to all-cause mortality while linearly related to cardiovascular mortality. Since lower ABI is a marker of atherosclerosis, it appears that lower ABPI values relate to a higher risk of mortality in adults without vascular stiffness.

Our study has several limitations. Firstly, older individuals only had leg blood pressure recorded once, which may result in some misclassification. Secondly, the retrospective study design, heart rhythm irregularities, high heart rate and differences in blood pressure between the arms and ankles may serve as limitations in explaining the causal relationship between ABPI and mortality.

## Conclusions

In our study, we found a lower ABPI was independently associated with an increased risk of all-cause and cardiovascular mortality in adults without arterial stiffness.

## Data Availability

The original data can be obtained from NHANES (https://www.cdc.gov/nchs/nhanes/index.htm).
